# Links of Consciousness, Perception, and Memory by Means of Delta Oscillations of Brain

**DOI:** 10.3389/fpsyg.2016.00275

**Published:** 2016-03-10

**Authors:** Erol Başar, Aysel Düzgün

**Affiliations:** Brain Dynamics, Cognition and Complex Systems Research Center, Istanbul Kültür UniversityIstanbul, Turkey

**Keywords:** top–down, P300, working memory, perception, consciousness, unconsciousness, brain oscillations

## Abstract

The aim of this report is threefold:

(1) First, we accomplish a survey integrating the description of consciousness, perception, and memory according to the views of descriptions of Hermann Helmholtz, Sigmund Freud, Henri Bergson, and Gustav Jung.

(2) In the second step, we present experimental results for defining the machineries of sensation and perception: (a) electrical responses of isolated ganglion of *Helix pomatia* were measured upon odor stimuli that elicited varied degrees of responses. Such a model may give an idea of the control of sensation in the preconscious state of a living tissue. (b) We also describe experiments at the human hearing threshold level. (c) Further, the omission of working memory will be shown with the attenuation of delta response in Alzheimer’s subjects in P300 measurements. (d) Finally, the measurement of auditory evoked potentials during slow-wave sleep in the cat brain explains the auditory responses that are not heard at this level of consciousness.

(3) In the third step, we aim to provide a synopsis related to *integration of perception, memory, and consciousness.* By using concepts of important scientists as S. Freud on consciousness, we also tentatively discuss the boundaries of the transition of unconsciousness states to conscious states.

## Introduction

### What Are the Main Principles of Perception, Memory, and Consciousness?

In the present paper, we aim to address an important chain of questions related to ground properties of the mindful brain. Several outstanding neuroscientists tried to describe machineries of cognitive processes and consciousness. However, the transition from unconscious to conscious states also merits special emphasis. Although most of the scientists realized the importance of the research accomplished by Sigmund Freud and Gustav Jung, none of the studies succeeded in exactly defining the boundaries of the conscious and unconscious states due to several reasons.

In the present paper, we aim to develop an essay including few steps analyzing the dynamic balance (equilibrium) between conscious and unconscious states. In order to approach this problem, we will use the rules of EEG-neurophysiology as well as the time frame needed during cognitive performances of the brain.

The physiology of the brain is anchored to important functions that also act in an integrative way: “Perception” and “Sensation”.

Several research scientists defined sensation as perception by means of sense organs. The philosopher Henri Bergson indicates “pure perception” as the simple reaction coming from sense organs. On the contrary, the perception can be defined according to Helmholtz as the integration of pure sensation with unconscious inference. Gustav Jung has analyzed the problem of consciousness by “integrating intuition,” “sensation,” “feeling,” and “thinking.” For Jung, expressing sensation is equivalent to perception. As feeling presently, psychologists prefer the expression “emotion”.

**FIGURE 1:**
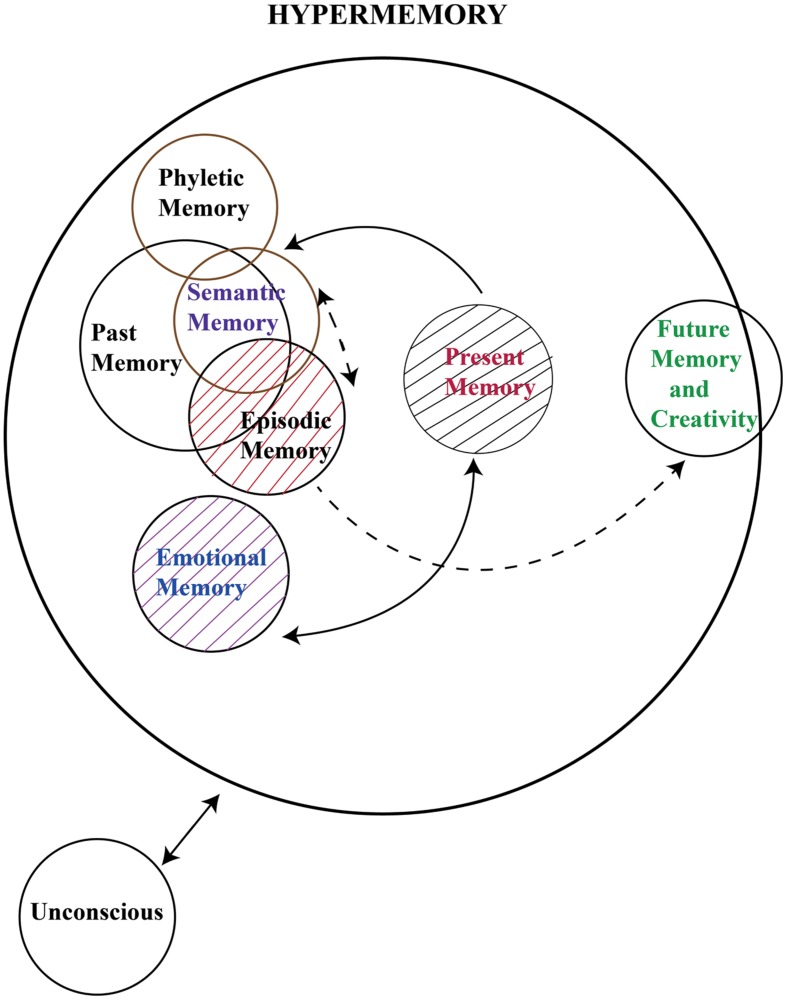
**In this illustration, past memory includes semantic memory and episodic memory.** Semantic memory and episodic memory are mostly overlapping in time, and possibly, they share similar neural networks. Emotional memory is also based on our past memory. Therefore, it is designed in an adjoining way to episodic memory. The illustration also indicates that present memory relies on past memory, and that there possibly are links from past memory to future memory and creativity. As stated by Bergson (1920), the consideration of future events requires past and present memories. As stated in the text, the time space, which we call hypertime space, requires a physical time period of approximately 0.5 s. In this illustration, the functioning of working memory and implicit memory are not yet incorporated. Modified and extended from Başar and Düzgün (2015).

According to Freud (Brown, 1914), the mind can be divided into three different levels:

1. The conscious mind includes everything that we are aware of. Examples are sensations, perception, and memory. A major part of this includes our memory, which is not always part of consciousness but can be retrieved easily at any time and brought into our awareness. Freud called this the “preconscious”.
2. The preconscious mind is the state of the mind that represents memory in the common sense. While we are not consciously aware of this information at any given time, we can retrieve it and pull it into consciousness when needed.
3. The unconscious mind is a reservoir of feelings, thoughts, urges, and memories that exists outside of our conscious awareness. Başar and Düzgün (2015) used the expression “Hypermemory” instead of “memories”. The concept of hypermemory is illustrated in **Figure 1**.

Most of the contents of the unconscious are unacceptable or unpleasant, such as feelings of pain, anxiety, or conflict. According to Freud, the unconscious continues to influence our behavior and experience, even though we are unaware of these underlying influences.

**FIGURE 2:**
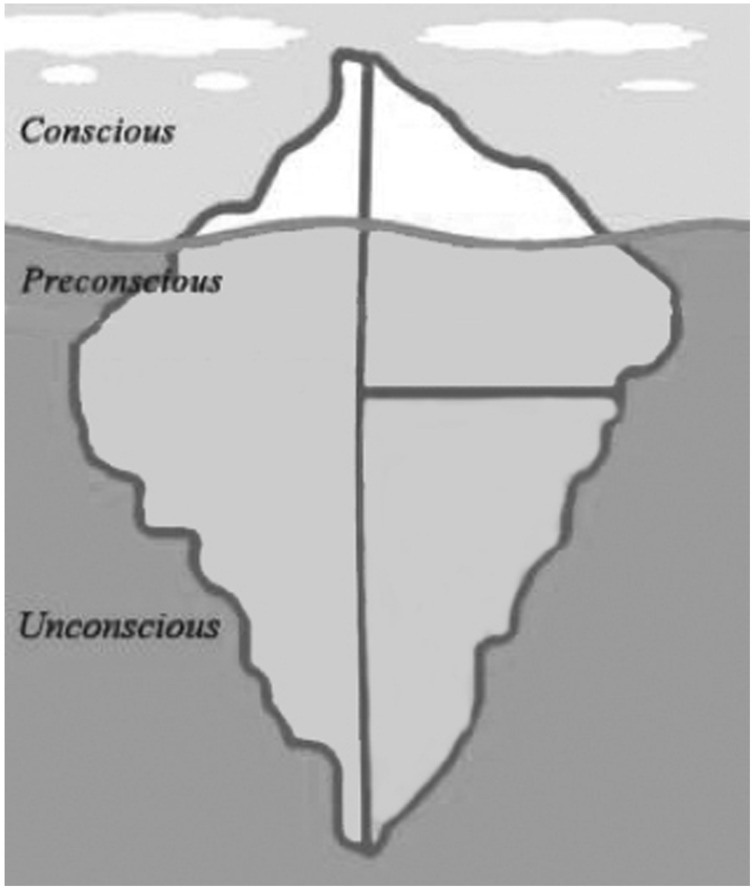
**Explanation see in the text**.

Freud likened these three levels of mind to an iceberg. The top of the iceberg that you can see above the water represents the conscious mind. The part of the iceberg that is submerged below the water but is still visible is the “preconscious”. The bulk of the iceberg lies unseen beneath the waterline and represents the unconscious (see **Figure 2**).

### View of Herman Helmholtz

Helmholtz (1867/1910) discussed the psychological effects of visual perception.

According to Helmholtz, the formation of visual impressions is achieved primarily by unconscious judgments, the results of which “can never once be elevated to the plane of conscious judgments” and thus “lack the purifying and scrutinizing work of conscious thinking.”

As the process is spontaneous and automatic, we are unable to account for just how we arrived at our judgments. Through our eyes, we necessarily *perceive things as real*, for the results of the unconscious conclusions are interpretations that “are urged on our consciousness (Helmholtz, 1867/1910).”

### Definitions of Gibson’s and Gregory’s Perception Concepts

In order to define sensations and perceptions to different processing:

Gibson (1966) has proposed a “direct” theory of perception, which is a bottom–up theory. On the contrary, Gregory (1970) has proposed a constructive undirected theory of perception expressed as “top–down” theory.

**FIGURE 3:**
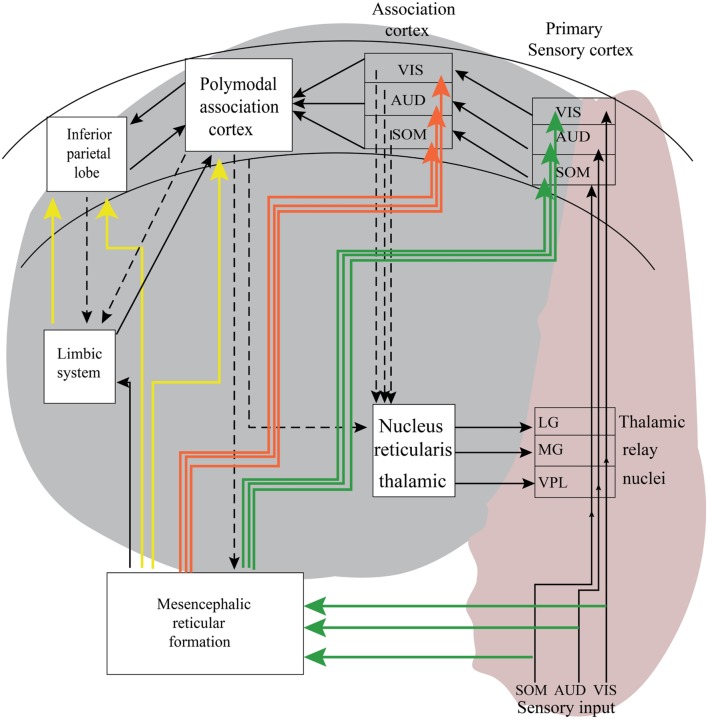
**The pink area in this schematic description of neural pathway is involved with bottom–up processing.** The gray areas in this illustration are tentative neural pathways, in which complex top–down processing may take place. As indicated with arrows during top–down processing, there are several possible loops generating recurrent reverberations. According to Fessard (1961), it is impossible to predict exactly in which areas the signal transmission ends. The exact duration of transmission is not possible, and this case is dependent on cognitive states. It is also possible to express that the results are probabilistic and also include the unconsciousness inference of Helmholtz.

**Figure 3** is a tentative presentation of neuronal networks to explain bottom–up and top–down processing. Sensations are elicited in the peripheral organs of the body, such as eyes, ears, and skin receptors. If we track visual information, the electrical impulses that are elicited in retina reach the occipital cortex (visual cortex), and travel through the visual pathway. These signals coming from the retina reach the visual cortex over thalamus (LG). This is a simple way of bottom–up processing. In real life, the subject’s cell receives pure sensory signals as a light stimulus. In general, more complex visual patterns contain elements from the history of subjects are presented. In **Figure 3**, there are also connections from the reticular formation and connections to the limbic system. Further, there are links from the visual cortex to other association areas of the brain. In the top–down processing, the signal flow in the brain hits several neuronal populations. This is a more complex signal processing, which usually ends in no predictable areas of the brain; therefore, this is a complex signal processing which shows uncertain reactions (see also Fessard, 1961).

## Working Memory: Oddball P300-Paradigm and Conscient Target Responses

In performing many complex tasks, it is necessary to hold information in temporary storage to complete the task. The system used for this is referred to as “working memory”(Baddeley, 1996). Working memory is the temporary ad hoc activation of an extensive network of short- or long-term perceptual component of that network would be, as any other perceptual memory, retrievable and expandable by a new stimulus or experience. Fuster (2013) states that working memory has the same cortical substrate as the kind of short-term memory traditionally considered the gateway to long-term memory.

The component analysis by means of event-related oscillations provide a real advantage over conventional ERP analysis as, for example, the results of cross-modality measurements demonstrate: in occipital areas, auditory stimulation does not evoke 10 Hz responses, although an ERP is measured upon visual stimulation. This demonstrates the dependence of the 10-Hz response on visual perception. Accordingly, the spatial resolution of ERPs is highly increased.

As to the delta response in the auditory P300 paradigm, a distributed highly enhanced response in the whole cortex is observed (Başar-Eroǧlu et al., 1992), the maxima being in frontal and parietal areas.

**FIGURE 4:**
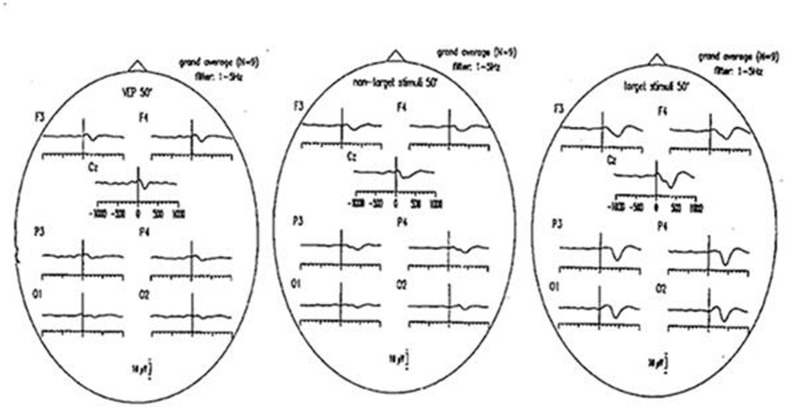
**Grand average ERPs (50 checkerboards; *N* = 9 subjects), filtered 1–5 Hz, respectively. (Left)** Visual evoked potentials (VEP), **(middle)** responses to non-target stimuli; **(right)** responses to target stimuli.

**Figure 4** illustrates visual evoked potentials with non-target and target responses as grand average of nine subjects. The delta responses filtered in the 1–3 Hz frequency window have largest values upon target stimuli. For further explanations related to delta responses we refer to (Schürmann et al., 1995).

### Working Memory in Mild Cognitive Impairment (MCI) and Alzheimer’s Disease (AD)

As the most common cause of dementias, Alzheimer’s disease (AD) is one of the most intensively researched subjects in neuroscience. AD is the most common and devastating cause of degenerative dementias and is generally found in people aged over 65. Approximately 24 million people worldwide have dementia, of which two-thirds are due to AD (Ferri et al., 2005). Clinical signs of AD are characterized by progressive cognitive deterioration, together with declining activities in daily life, and by neuropsychiatric symptoms.

Delta responses of AD patients are almost completely abolished, whereas in mild cognitive impairment (MCI) patients, delta responses are gradually decreased.

**FIGURE 5:**
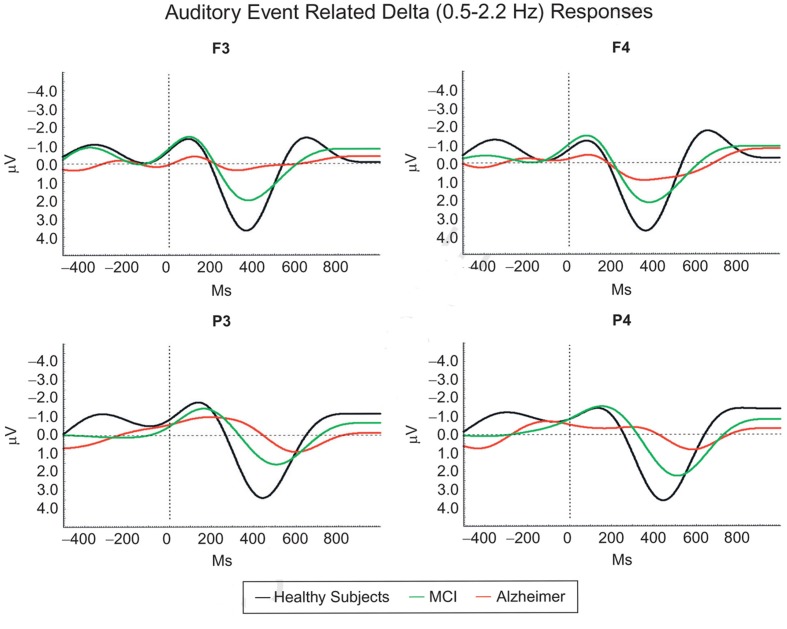
**Mild cognitive impairment (MCI) and Alzheimer’s disease (AD) continuity is prominent in auditory event-related delta oscillatory activity, showing gradually decreasing delta amplitudes and delayed delta peak responses among healthy subjects, MCI, and mild Alzheimer subjects (Modified from Yener et al., 2011)**.

As seen **Figure 5**, in AD patients that are not capable of performing cognitive functions, the late delta response is gone. The explanation is as follows:

The top–down processing does not take place or it is attenuated. It is further noted that AD-diseased frontal lobes or hippocampus have lesions. The anatomical evidence indicates that the cognitive circuitry is partially out of function.

## How Can We Find a Neural Tissue Showing Only Sensory Responses (Phyletic Responses)?

We are in search of a biological model that does not perform a cognitive function but does perform a pure sensation. We have performed measurements with the isolated ganglion of *Helix pomatia* (snail), which responds with increased electrical activity upon electric stimulation. This is possibly due to the machinery of electrical susceptibility, which can be recorded *in vitro*. Do we have another possibility to find an isolated tissue that responds to sensory stimulation? Such a model was published by Schütt et al. (1999), who measured responses of a pedal ganglion of *H. pomatia* (snail) to different odorous stimulations.

**FIGURE 6:**
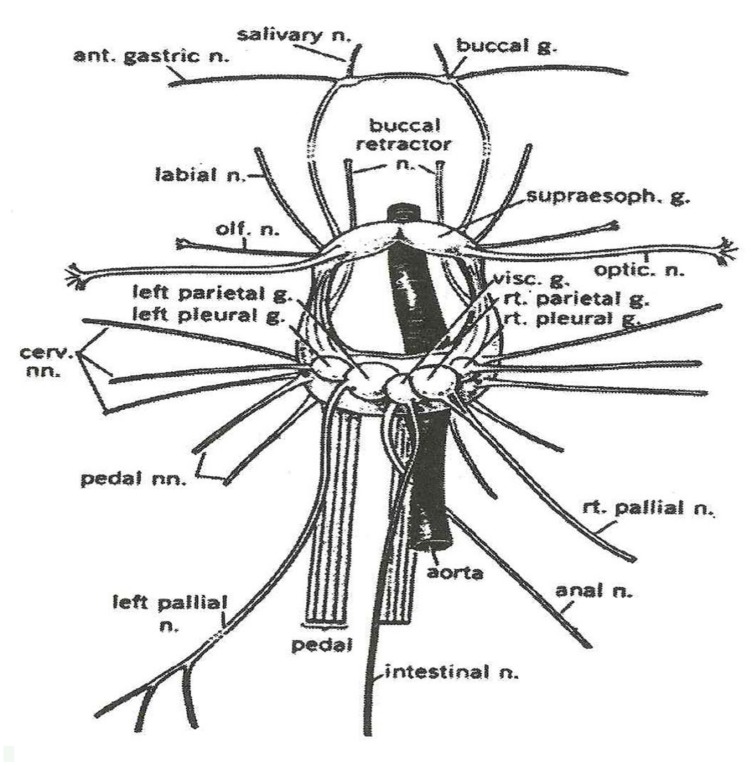
**The central nervous system and major nerves of a young *Helix aspersa* (Modified from Courtesy G. Kerkut)**.

**Figure 6** illustrates the snail ganglion, which is composed of several almost identical neurons.

Ganglia of *Helix* are composed from approximately 2000 neurons.

**FIGURE 7:**
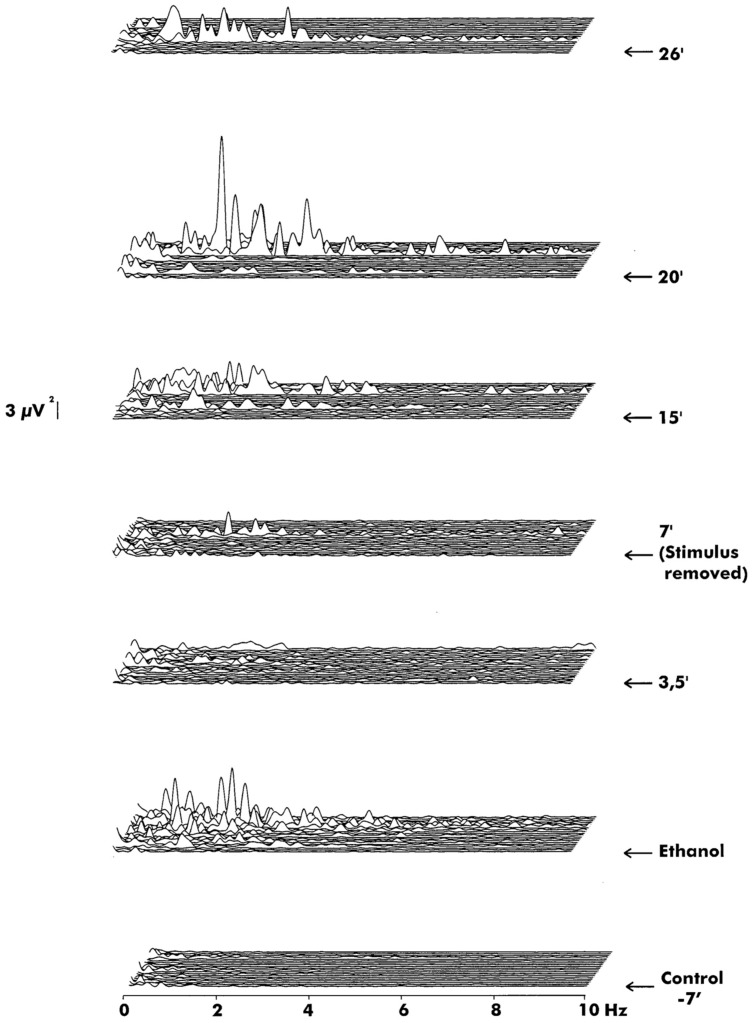
**The power spectra of recordings from the corresponding experiment.** Each panel consists of 20 single epochs (204.8 s). From bottom: control (prestimulus), immediately after application of ethanol and the following recordings at different times. The stimulus was removed at 7 min after stimulus onset. Note that fluctuation with multiple frequency components reaching well into 10 Hz and beyond was induced shortly after ethanol, most strongly in the 0.5–5 Hz range (panel 2). Then it subsided to occasional small bursts (panels 3 and 4). Firing continued intermittently even after removal of stimulus (panels 5–7).

**Figure 7** shows different reaction patterns to an odorous stimulation. The bottom–up aspect of the stimulation is power spectral activity under control. In the second step, ethanol stimulation activates the *Helix* ganglion, and power spectra show great responsiveness that declines following removal of stimulation.

**FIGURE 8:**
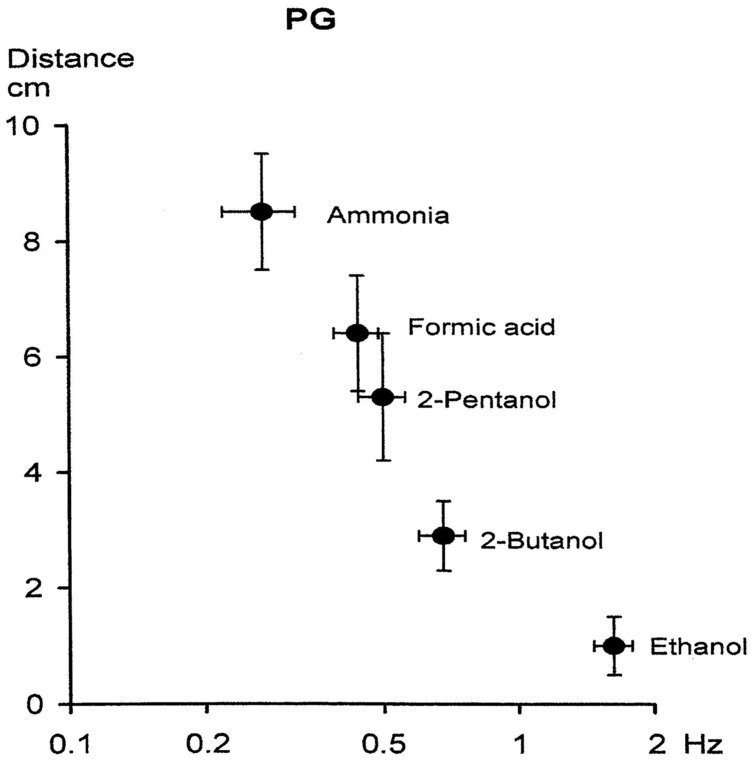
**Relationship between degree of aversion and frequency of odorant-induced activity of the *Helix* pedal ganglion.** The bars represent 95% confidence limits for the means. Note that there was a linear relationship between degree of averseness and peak power frequency of odorant-induced FP activity in the *Helix* ganglion. This diagram suggests: (a) order of averseness appears to reflect that of molecular affinity: the stronger in affinity an odorant, the more aversive it is to the snail; (b) order of affinity is correlated to peak power frequency of induced FP: the stronger in affinity an odorant, the lower the odorant-induced frequency; (c) the more aversive an odorant, the lower the peak power frequency; (d) extrapolation of the curve to the abcissa yields a value of ∼2.5 Hz. This is the area for an appetitive odorant (ethanol) for the snail. These curves suggest that the odorant-specific low frequencies may, together with other frequency components, be involved in identification, classification, and discrimination of odorants or their classes and that the most crucial FP activities relevant for this function may exist below ∼2.5 Hz (Modified from Schütt et al., 1999).

The increase in the concentration of ethanol elicits higher power spectral peaks in the delta frequency ranges, similar to the reaction of ethanol. The formic acid also elicits high amplitude reactions in the delta frequency range similar to the reaction of formic acid. Phentonol also induces higher spectral responses in the delta frequency range. **Figure 8** illustrates the effects of distance of *Helix* delta response to various odor stimuli.

The most ample odor, ammonia, elicits the highest delta responses. This is a crucial point. According to our experience, human control subjects are most excited with the odor of ammonia, which almost always elicits a very unpleasant sensation. This comparison demonstrates the great similarity between sensations of human beings and of isolated unconscious (preconscious) *Helix* ganglion. Possibly, it can be assumed that the delta responses are manifestations of preconsciousness and consciousness reactions in living beings. Is it possible to say that the transition from unconsciousness states is performed in the delta frequency range? We confine our attention to this odorous stimulation and describe delta frequency range. However, we tentatively pronounce that the slow oscillations possibly can solve a pivot function during transition to the different states of consciousness.

## How to Measure Perception at the Human Hearing Threshold?

**FIGURE 9:**
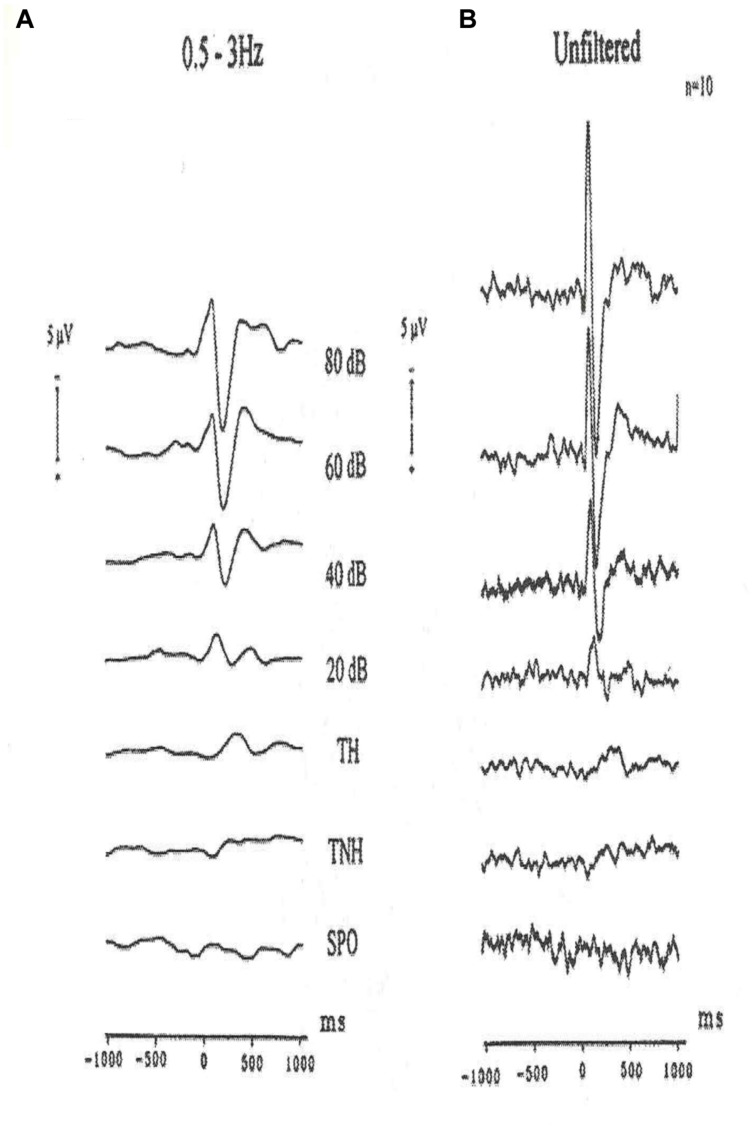
**Grand average auditory evoked potentials recordings of ten subjects (vertex). (A)** 0.5–3 Hz digitally filtered AEPs to different stimulation intensities. TH (tone heard) and TNH (tone not heard) are subgroups of the threshold experiments. SPO are the spontaneous activity experiments. **(B)** The same potentials are reproduced unfiltered. Negativity is upward.

In this section, we confine our attention to another type of problem. We consider a paradigm that encompasses measurement of a process to detect sensations and judge sensations:

How to measure perception at the human hearing threshold model? We report here measurements performed by Parnefjord and Başar (1999) by application of auditory stimuli at the hearing threshold level. The subjects sat in a dimly illuminated and 120 dB isolated room and were exposed to auditory stimuli at different tone levels. The measurements have started with 80 dB signals and were step-wise decreased first to 20 dB level and finally to the hearing threshold level. In **Figure 8**, the unfiltered evoked potentials and filtered evoked potentials in the delta frequency range are presented. The measurements of Parnefjord and Başar (1999) have shown that at the threshold level of all oscillatory responses besides delta response disappeared (**Figure 9**).

At the hearing threshold level, the remaining response is the delta response. These facts are also seen in the unfiltered evoked potentials. These authors have interpreted that the responses are correlated to simple sensation, threshold perception, and decision making. In other words, in comparison to experiments on *H. pomatia*, the response is correlated with cognitive tasks. However, the cognitive task does not contain working memory task, which is performed in the target response during the P300 experiment, (see Parnefjord, 1996).

**Figure 9** shows the results of experiment with single subjects in different frequency windows for the wide band evoked potentials at 80 dB and at the hearing threshold level.

**FIGURE 10:**
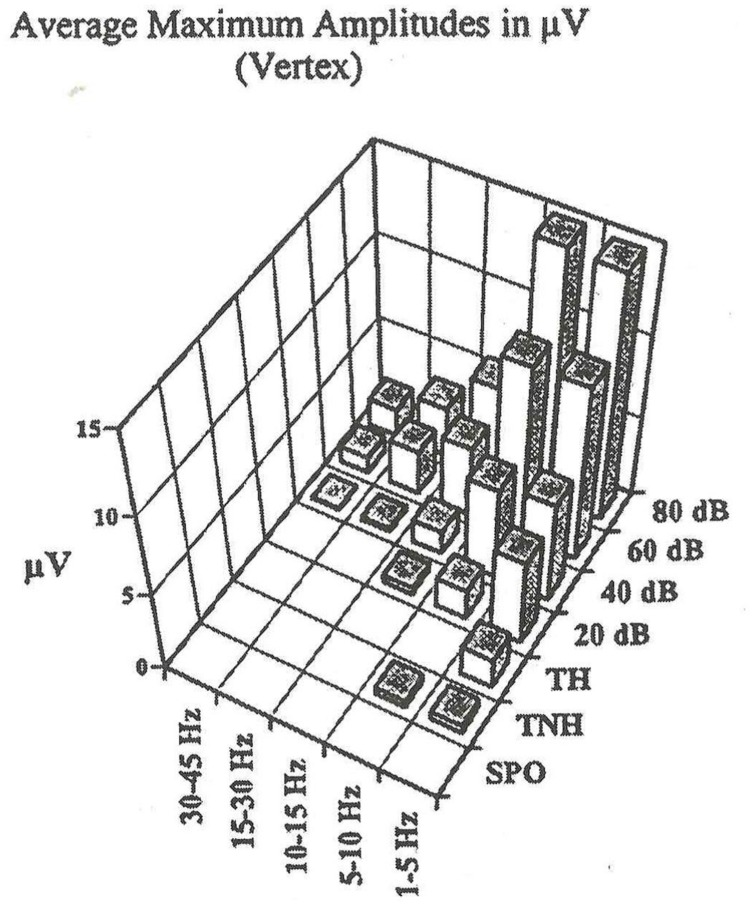
**Grand average peak-to-peak AEP amplitudes of ten subjects (vertex recording).** Below are the absolute values, together with standard deviations, above a histogram representation. TH; TNH: both subgroups of threshold stimulation. SPO = spontaneous activity experiments. The evoked potentials to the different stimulation intensities have been digitally filtered in different frequency ranges. The resulting peak-to-peak amplitudes of the digitally filtered activities in different frequency ranges. The resulting peak-to-peak amplitudes of the digitally filtered activities are presented.

**Figure 10** shows the following: At the hearing threshold level the remaining responses has only a delta oscillatory component. Other higher frequency response oscillations are gone.

## Auditory Evoked Oscillations and Coherences During Slow-Wave Sleep of the Cat

**FIGURE 11:**
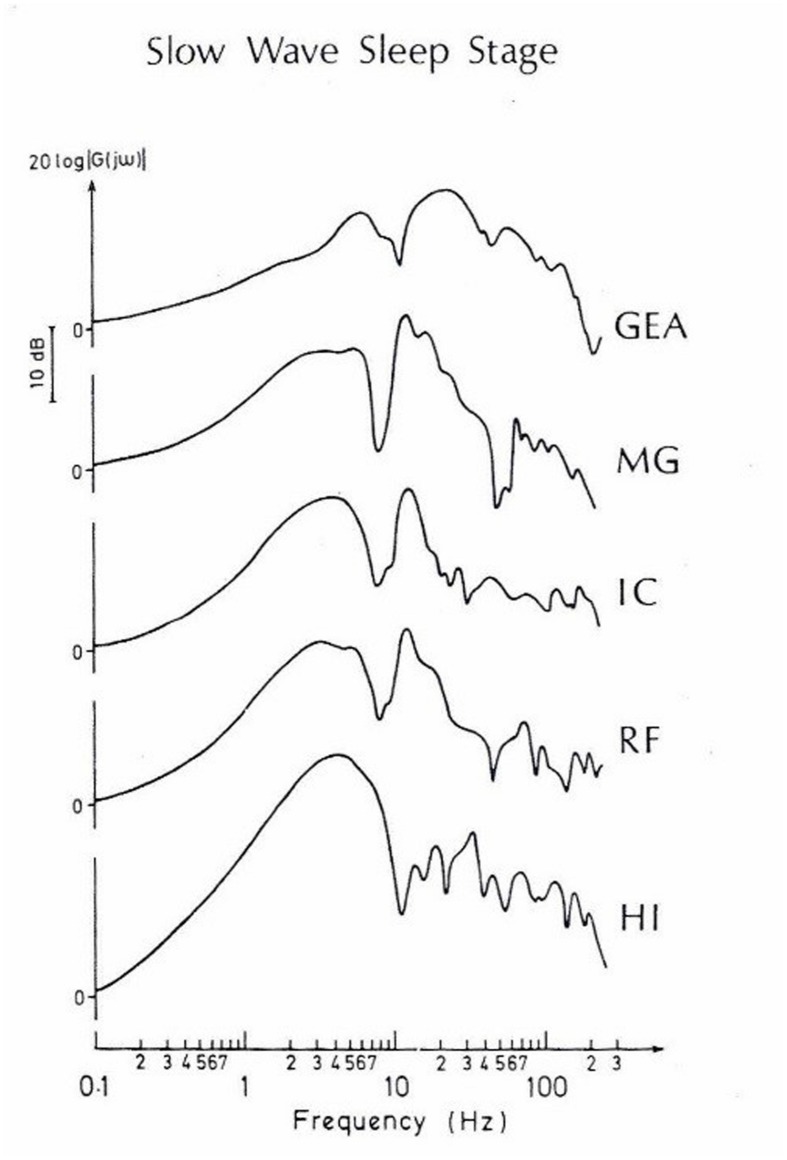
**A typical set of amplitude-frequency characteristics obtained by means of the frequency response analyze method and using the selectively averaged evoked potentials (SAEPs), which were simultaneously recorded from different brain nuclei of the cat during the slow-wave sleep stage.** Direct computer plottings. Along the abscissa is the input frequency in logarithmic scale, along the ordinate is the potential amplitude, | G(jω)|, in decibels. The curves are normalized in such a way that the amplitude at 0 Hz is equal to 1 (or 20 log 1 = 0). (Modified from Başar et al., 1979).

**FIGURE 12:**
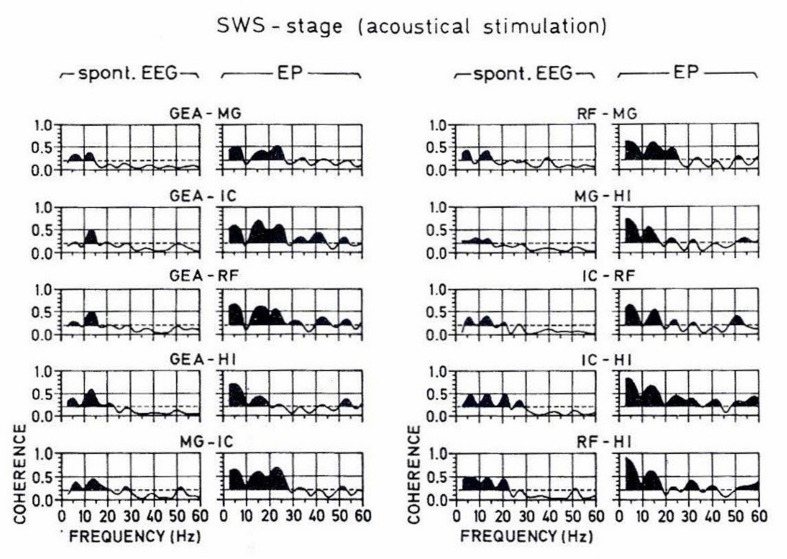
**A typical set of coherence functions computed from the spontaneous and evoked potentials of all possible pairings of the studied brain structures during the slow-wave sleep stage.** The scale is indicated at the bottom. Along the abscissa is the frequency from 0 to 60 Hz, along the ordinate is the coherency between 0 and 1. The horizontal broken lines indicate the significance level, which is 0.2 for all plots. The area under the coherence functions is darkened only if the curve surpasses this level. In order to facilitate a comparison between the coherence values computed from spontaneous and evoked parts of the EEG, the respective coherence functions are presented adjacently as couples for all the pairings of recording electrodes (From Başar et al., 1979).

During slow-wave sleep, evoked spectra, and the connectivity between intracranial structures of the cat brain are also changed (see **Figure 11**). The spatial coherency is presented in **Figure 12**, in which coherences between these five structures of the brain are shown. Here again, the coherence functions reach maximal strength in the delta frequency range, dominantly in deeper structures as reticular formation, hippocampus, and thalamus. The simple interpretation of coherence results show that during SWS sleep, the connectivity is performed mostly in the delta frequency channel. In other words, the cat in the deep sleep stage does not perform conscious hearing, but the electrical processes further take place dominantly in the delta frequency range.

## A Synopsis Based on Multiplicity of Percepts

**FIGURE 13:**
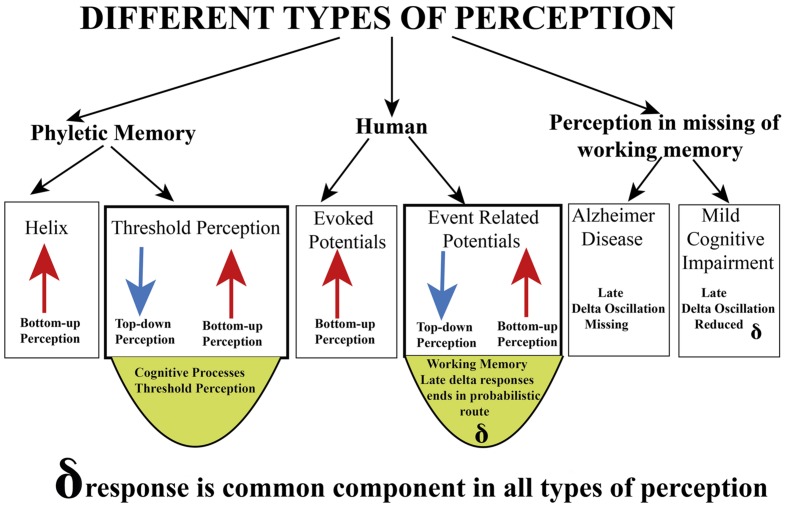
**Delta responses in different types of perception**.

(1) In the present comparative study, we described five major experimental designs in order to establish a progressive synopsis for the understanding of perception and perception-related concepts. Further, as we have analyzed in previous sections, it is not possible to isolate the concept of perception without combination of attention, memory, and learning processes. Further, the concept of unconscious inference by H. Helmholtz should be considered as an important enrichment to episodic memory.
In **Figures 7** and **8**, we explained experiments with an isolated pedal ganglion of *Helix* and assumed that this ganglion *in vitro* is a tissue that is in a preconscious state, but not in a conscious state since this odorous stimulation is not processed as odor. This ganglion behaves as an odor sensitive organ that is not able to perform cognitive functions, or in other words, not able to transfer the odor sensation to a conscious process. The ganglion does not have the ability to consciously differentiate the degree of odor sensations or the quality of sensations, but certainly the ganglion *in vitro* can differentiate odors without being aware of this basic function, which could be also denoted as phyletic memory. We have to emphasize that this tissue is an example showing only *phyletic memory*, and this memory cannot be influenced or altered by unconscious inference.
(2) After comparing different experimental designs shown in **Figure 10**, we can experimentally differentiate several perceptions and “perception-action cycles”. Delta response as a major component. We also emphasize that delta oscillations are correlated at least with two different functions:
Delta in the first 200 ms is a bottom–up process. Beside this, the late delta response in the oddball experiment is a cognitive perception upon increased attention in a working memory process. It is also mentioned that, vice versa, delta component is participating in two different types of processes.
In **Figure 13**, we also compared evoked potentials and event-related potentials upon extended stimuli in multiple areas. It is known that one of the major components of the oddball P300 response is “delta response” measured 400 ms following target stimulations. In the recordings of sensory-evoked potentials, upon simple light stimulation, no late delta response is observed. In the first 200 ms, a preliminary delta response is recorded upon simple light. Here it is noted that cognitive processing requires a longer time period. Further, the absence of the delta response in the simple evoked potentials indicates almost a pure bottom–up response in the first 200 ms. In other words, by comparing the delta response in simple evoked potential and in the target response, it is possible to separate the top–down and bottom–up components (**Figure 3**). The next step: will it be possible to find a situation or an experimental procedure to further support this statement?
(3) Third step (in **Figure 13**) involves an experimental design to measure the oscillatory responses at the human hearing threshold level. In this experiment, the subject attentively hears an auditory stimulation at the threshold level, and the subject is involved with a hearing process and following the hearing, he has performed the cognitive processes of “threshold perception” based also on a “decision making process”. Accordingly, auditory delta response in this experiment highly differs from the delta response of the *Helix* ganglion to odorous stimuli. The subject is conscious about the heard tones whereas the *Helix* delta response is most probably a “preconscious” process. Further, the auditory delta response at the hearing threshold is result of an activation of bottom–up and top–down networks (see **Figure 3**). We also compare sensory delta responses and target delta responses in MCI and AD patients.
Delta response of the isolated *H. pomatia* ganglion can be considered as a preconscious (unconscious) state-response.
(4) Delta response in slow-wave sleep. During SWS, we observed phase-locked delta oscillations and increased delta connectivity. This delta-response is a manifestation of sensory processing in an unconscious state (deep sleep). The auditory stimulation is not consciously heard by sleeping animals. The hearing threshold is very high in SWS sleep.

## Conclusion

In conclusion, we emphasize the following points:

(1) Delta response is recorded during P300 Oddball paradigm as response to target stimuli, indicating the manifestation of working memory. The conscious observation of light stimuli is absent in AD patients. Accordingly, conscious detection of cognitive stimulation disappeared.
(2) Non-conscious *H. pomatia* Pedal Ganglion is also able to manifest a delta power increased to different odorous stimulation.
(3) At the human threshold level, the heard tones also manifest with a dominant delta response. Other frequency responses are absent.
(4) During slow-wave sleep, ample acoustic stimuli of 80 dB elicits a large delta response. Further, the delta coherency between long distance structures of the cat brain shows highly increased delta components. In other words, during deep sleep, the delta response is existent, although the cats cannot consciously hear the auditory stimuli.

According to this observations, the following concept could be tentatively addressed:

“Does the delta response manifest a transition through the gates between conscious, preconscious, and unconscious states?

In this report, we used the modifications of percepts at elementary level. As we have previously described for other EEG frequency components are also important for generation of percepts. However, for this report, we used only delta activity at the conscious level. Descriptions of all other components cannot be described in a short paper with limited volume.

## Author Contributions

The authors EB and AD have jointly analyzed the outcome of earlier performed and published experiments. The comparisons and final conclusions related to transition between conscious and unconscious states were developed and written together by EB and AD.

## Conflict of Interest Statement

The authors declare that the research was conducted in the absence of any commercial or financial relationships that could be construed as a potential conflict of interest.
